# Effects of Plyometric Jump Training in Sand or Rigid Surface on Jump-Related Biomechanical Variables and Physical Fitness in Female Volleyball Players

**DOI:** 10.3390/ijerph182413093

**Published:** 2021-12-11

**Authors:** Mina Ahmadi, Hadi Nobari, Rodrigo Ramirez-Campillo, Jorge Pérez-Gómez, Alexandre Lima de Araújo Ribeiro, Alejandro Martínez-Rodríguez

**Affiliations:** 1 Department of Exercise Physiology, Faculty of Sport Sciences, University of Isfahan, Isfahan 81746-7344, Iran; minaahd7@gmail.com; 2 Department of Exercise Physiology, Faculty of Educational Sciences and Psychology, University of Mohaghegh Ardabili, Ardabil 56199-11367, Iran; hadi.nobari1@gmail.com; 3 Department of Physiology, School of Sport Sciences, University of Extremadura, 10003 Cáceres, Spain; 4 HEME Research Group, Faculty of Sport Sciences, University of Extremadura, 10003 Cáceres, Spain; jorgepg100@gmail.com; 5 Departamento de Ciencias de la Actividad Física, Universidad de Los Lagos, Santiago 8320000, Chile; r.ramirez@ulagos.cl; 6 Exercise and Rehabilitation Sciences Laboratory, School of Physical Therapy, Faculty of Rehabilitation Sciences, Universidad Andres Bello, Santiago 7591538, Chile; 7 Physical Education College, University of Brasilia, Brasilia 70910-900, Brazil; alexandrelaribeiro@gmail.com; 8 Department of Analytical Chemistry, Nutrition and Food Sciences, University of Alicante, 03690 Alicante, Spain; 9 Alicante Institute for Health and Biomedical Research, ISABIAL, 03010 Alicante, Spain

**Keywords:** sports medicine, human physical conditioning, resistance training, strength training, sports

## Abstract

Background: This study aims to assess the effects of 8 weeks of plyometric jump training (PJT) conducted on sand or a rigid court surface on jump-related biomechanical variables and physical fitness in female indoor volleyball players. Methods: Seventeen participants were randomly divided into a sand surface group (SsG, *n* = 8) and rigid surface group (RsG, *n* = 9). Both groups completed equal indoor volleyball training routines. Participants were assessed pre and post the 8-week PJT for jump-related biomechanical variables (countermovement jump (CMJ) RSI; drop jump (DJ) reactive strength index (RSI); spike jump (SJ) height; CMJ height; CMJ rate of force development (RFD); CMJ velocity at take-off; DJ height and CMJ peak force), 20 m linear sprint time, *t* test for change-of-direction sprint (CODs) time, Wingate test peak power (PP), cardiorespiratory endurance, and leg-press one-repetition maximum (1RM). Results: A two-way mixed analysis of variance (group × time) revealed that there was a significant group × time interaction between DJ height (*p* = 0.035) and CMJ peak force (*p* = 0.032) in favour of RsG and SsG, respectively. A significant interaction was also observed for cardiorespiratory endurance (*p* = 0.01) and 1RM (*p* = 0.002), both favouring the SsG. No other group × time interaction was observed. Conclusions: The type of surface used during PJT induced specific adaptations in terms of jump-related biomechanical variables and physical fitness in female indoor volleyball players. Based on the individual needs of the athletes, practitioners may prescribe one type of surface preferentially over another to maximize the benefits derived from PJT.

## 1. Introduction

A volleyball match alternates between short periods (i.e., 3–9 s) of maximal intensity and relatively long recovery periods (i.e., 10–20 s) [1]. Thus, physical fitness variables such as frequent sprinting, change-of-direction speed (CODS) and anaerobic power can influence a volleyball player’s performance [2]. Additionally, cardiorespiratory endurance helps to reduce recovery time after maximal intensity efforts during a total match time of nearly 60–120 min [1]. Among physical fitness traits, volleyball players’ jumping ability is crucial to scoring and defending points due to the characteristics of the game [3]. According to the training principle of specificity, plyometric jump training (PJT) may be an important element in a volleyball player’s regular training schedule [4]. A systematic review indicated that PJT increased volleyball players’ strength, agility/speed and vertical jump performance [5], potentially helping players during decisive match scoring point actions (serve, spike, and block).

Several PJT variables can be manipulated to increase PJT effectiveness, such as volume, intensity, and the type of jump [6]. However, environment-related variables, such as the type of surface (e.g., sand, natural and artificial grass), should also be considered during PJT programming [7,8]. Among team sport athletes, the optimal type of surface for PJT has not been identified. To date, a few well controlled studies have examined the potential effects of different PJT surfaces on components of physical fitness and explosive performance in athletes. Impellizzeri et al. (2008) investigated the effects of a 4-week PJT on sand vs. grass surface on the vertical jump height and 10 m and 20 m linear sprint ability of amateur soccer players. The results indicated that sand induced greater squat jump improvements (9.25%) compared to grass (5.02%), and a greater improvement in 20 m sprint time was seen for grass (27.86%) compared to sand (25.07%) [9]. A previous study, conducted by Arazi et al. (2014), examined the effects of PJT on sand vs. land surface and confirmed significant improvements in vertical jumping and 20 m sprint time after land-based PJT and CODS and leg-press one-repetition maximum (1RM) after sand-based PJT in healthy men following 6 weeks of treatment [10]. Ramirez-Campillo et al. (2013) noted greater improvements in maximal dynamic strength, jumping and sprinting among physically active adolescent males after 7 weeks of PJT conducted on a higher-impact reaction force surface compared to a lower-impact surface [11]. Very recently, Ramirez-Campillo et al. (2020) examined the effects of 8 weeks of combined-surface PJT (grass, land-dirt, sand, wood, gym mat, and tartan-track) vs. single-surface PJT (grass) on physical fitness components in male adolescent soccer players. Although both training modalities reported significant improvements in countermovement jump (CMJ), drop jump from 20 cm (DJ20), CODS and 30 m sprint time, the advancements were in favour of combined surfaces during PJT [8]. Nevertheless, to emphasize the importance of spike jump heights, the strong contribution for predicting spike jump heights has been previously found with the F–T curve variables derived from CMJ (71%). It has been previously found that F–T curve assessments show that the force, velocity, power, rate of force development (RFD), and modified reactive strength index (RSImod) are the best predictors of SJ height in young elite volleyball players [12].

However, the aforementioned studies [8,9,10,11] included athletes with different sporting and fitness backgrounds (e.g., soccer; recreative trained), potentially making the results inapplicable for a particular sport such as volleyball. Indeed, the adaptive responses to PJT may be affected by moderators such as training background [13,14]. To our knowledge, only two studies have addressed the effects of the type of surface during PJT on volleyball players. Cimenli et al. demonstrated similar jump height improvements in male volleyball players after PJT conducted on a wooden and synthetic surfaces [15]. Another study on young male volleyball players observed a greater vertical jump height improvement following a 2-week PJT in sand compared to a rigid surface [16]. However, none of the aforementioned studies (including those on non-volleyball athletes) involved female participants. As the effects of PJT may be moderated by the sex of the participants, particularly for jump-related outcomes [17], there is a lack of research regarding training surfaces for female volleyball players; an investigation into this could assist coaches in manipulating conditioning training according to principle of training, and it would also offer novel and relevant insights on the topic. Therefore, this study aimed to assess the effects of 8 weeks of PJT conducted on sand or a rigid court surface on jump-related biomechanical variables and physical fitness in female indoor volleyball players. Based on the relevant literature [15,18,19], we hypothesized that PJT in either sand or a rigid court surface would improve jump-related biomechanical variables and physical fitness in female indoor volleyball players, with greater improvements after sand-based PJT.

## 2. Materials and Methods

### 2.1. Participants

**Figure 1:**
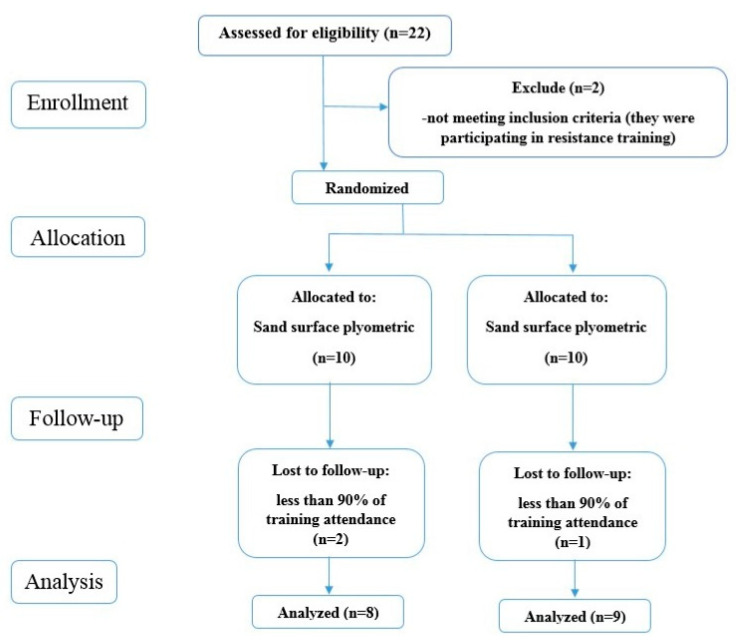
CONSORT diagram of participants’ recruitment, allocation, follow-up and.

Twenty-two female volleyball players agreed to participate in this study, and the athletes’ characteristics are presented in Table 1. To be included, the athletes had to meet the following criteria: (i) have at least three years’ experience in volleyball training and competition; (ii) take part in regular volleyball training and competition for five months prior to this study; (iii) have no illnesses or musculoskeletal injuries limiting their systematic training experience in the six months prior to this study; (iv) no participation in any other resistance or PJT programs outside this study; and (v) no surgery for two years prior to this study. Additionally, the athletes were excluded if: (i) they missed two or more PJT sessions; (ii) they missed any of the testing sessions. Due to these criteria, two athletes were excluded. The remaining participants were randomly assigned to a sand surface group (SsG, *n* = 10) or a regular volleyball rigid surface group (RsG, *n* = 10). However, due to low PJT attendance, the final sample was *n* = 8 for SsG and *n* = 9 for RsG. The CONSORT diagram of participants’ recruitment, allocation, follow-up and analysis is indicated in Figure 1. The ethics committee from the University of Isfahan (IR.UI.REC.1398.097) approved this study, and the recommendations for Human Ethics in Research were followed according to the Helsinki Declaration. Athletes received a clear explanation of the study, including all procedures, potential risks, and benefits involved, and signed their written informed consent before baseline assessments.

### 2.2. Design

To test our hypothesis, we used a randomized quasi-experimental study design with parallel groups and 8 weeks of PJT, with pre- and post-training periods. Participants were randomly allocated to the experimental group (*n* = 10) using the method of randomly permuted blocks (available at http://www.randomization.com, accessed on 1 August 2019). Note that this study was conducted during the pre-season, with athletes completing three training sessions per week [20] and a friendly competition match on weekend days.

### 2.3. Experimental Procedures (PJT Program)

The PJT was performed for 8 weeks, twice per week (Sunday and Wednesday). Table 2 shows that the total number of ground contacts per week increased progressively from 72 to 120 [21], and that each PJT session included 6 to 10 jump exercises, involving SSC muscle activity and arm swing. All PJT sessions lasted for approximately 40 min and were performed just after the warm-up, which involved jogging, dynamic movements of skips and lunges, and dynamic stretching of the lower-limb muscles [21]. Both groups completed the same number of total jump repetitions during the intervention on different surfaces. The SsG used sand, while the RsG used a wooden surface common in indoor volleyball courts. All PJT sessions were performed in the morning and athletes were asked to put in maximal effort throughout the jumps.

### 2.4. Outcome Assessments

The athletes were considered highly familiarized with testing protocols, since they had ≥3 years of background experience in similar testing procedures as part of their regular training preparation. All testing sessions were carried out at the same time of day. The athletes were instructed to attend testing sessions with the same sports shoes they used in matches and avoided intense physical activity during the days preceding testing, and the team staff programmed low-intensity technical-tactical drills during the testing week.

An experienced strength and conditioning coach, blinded to the study’s aim and participant group allocation, conducted all testing procedures, which were carried out over five days, with approximately 24 h of rest between each session. The same procedures were performed at pre and post intervention. On day one, anthropometric (body mass and height were recorded to the nearest 0.1 kg and 0.01 m using an electronic scale (SECA, Instruments Ltd., Hamburg, Germany)) and jumping tests were performed. On the second day, the 1RM leg press test was carried out. On the third day, athletes performed 20 m linear sprint and CODS tests. On the fourth day, the Wingate test was carried out. Finally, on day five, cardiorespiratory endurance performance was assessed. Before each testing session, the athletes completed their typical general warm-ups (10 min of walking/jogging and light stretching, followed by 10 min of more intense dynamic activity (skipping, leg swings, arm swings)), plus a specific warm-up according to the test of the day. Thereafter, the players had 3–5 min of rest before starting the tests. In the 1RM test, the Wingate tests, and the cardiorespiratory endurance test, only one maximal attempt was performed. The other tests involved three valid maximal attempts, and the best result was used for analysis. Five minutes of active rest (low-intensity volleyball drills) was allowed between attempts.

Athletes’ trials were recorded with a sampling of 1000 Hz using a Kistler force plate (500 × 600 mm, model AA 9260; Kistler, Switzerland), in line with a three-dimensional motion analysis system with seven cameras (Qualisys Track Manager, version 7.2, Sweden, Göteborg, Kvarnbergsgatan). Athletes were instructed to one second stand of the data collection moment to determine their body weight. Thereafter, a minimum 5 s data-recording window was considered for all jumping measurements. We also emphasize that an experienced laboratory technician supervised all valid attempts and the intra-class correlation coefficient (ICC) for all jumps was 0.87 to 0.96. For the analyses of these data, raw vertical force–time (F–T) data were exported as text files and graphical analysis was carried out with Excel software (version 2016, Microsoft Corp, Redmond, WA, USA).

#### 2.4.1. Drop-Jump Tests (Height, RSI)

In this study, we adopted the DJ35 to assess the DJ performance. The participants were instructed to drop down from a 35 cm drop box on the force platform, followed by a vertical jump. Athletes were asked to minimize ground contact time to reach the maximal vertical height [22].

The RSI was determined during the DJ as the ratio between jump height and time spent in contact with the ground as follows [23]: RSI = jump height (meters)/ground contact time (seconds).

#### 2.4.2. Spike Jump Test (Height)

Afterwards, athletes were instructed to perform a spike jump (SJ) using a two- or three-step approach to jump through the force-plate area (a 3 m-wide corridor) with hitting the ball at the highest possible height. To ensure constant testing conditions, the ball was held by the coach while standing on a chair (it was placed at the end of corridor) [12]. A three-dimensional motion system with 8 cameras was used to measure the kinematic parameters. Tow reflective markers were placed “on the iliac crest”, which is one of the points that is defined as an anthropometric reference point, to measure the whole body centre of mass (COM). The SJ height test was based on previous recommendations, and the COM height difference from the standing position to the maximum point achieved in the jump was calculated [12].

#### 2.4.3. Counter Movement Jump Test (Peak Force, Height, RSI, RFD)

The athletes were instructed to execute a counter movement jump (CMJ) on the force plate to a self-selected depth as fast as possible to achieve the maximal jump height, whilst using arm swing to mimic a real-game volleyball performance (interspersed with approximately one minute of rest) [12]. Peak force was measured as the maximum force generated before take-off [12,24].

Vertical acceleration was calculated using F–T curves divided by body mass. The vertical velocity of the COM was calculated by employing the trapezoidal integration with respect to time. Thereafter, CMJ height displacement was assessed by integrating velocity data with respect to time [25].

The reactive strength index for CMJ was calculated as previously suggested (i.e., jump height (m) divided by time to take-off (s)) [12].

**Figure 2:**
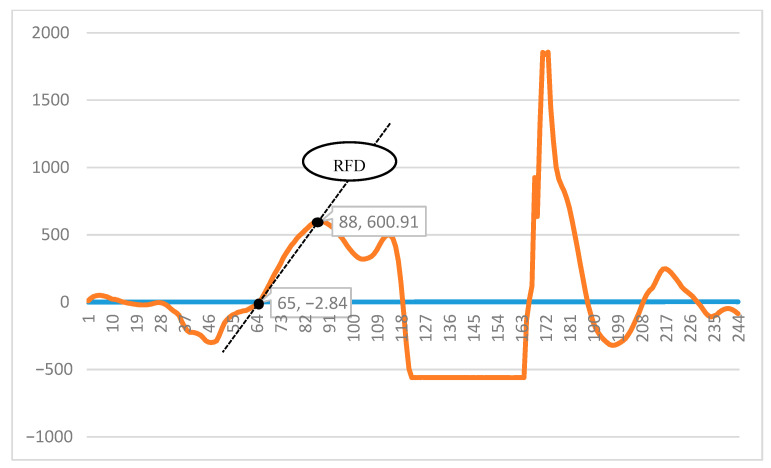
Representation method used to quantify average rate of force development (RFD) from the force–time curve.

The F–T curve of CMJ included eccentric and concentric phases [12]. The average RFD was determined as the maximum produced concentric force, which was divided by the time taken to reach peak force, difference (Δforce/Δtime (s)) in the slope of the ground reaction force–time record [26] which is indicated in Figure 2.

#### 2.4.4. RM Leg Press Test

The bilateral concentric leg press test was used for the 1RM test [27]. The test was performed with standard equipment (Gym Tech), and the athletes positioned themselves in a seated position, with 90° of knee flexion and 120° of hip flexion, and had to exert maximal concentric effort to reach full knee extension (i.e., 180°). Before this test, the athletes performed a specific warm-up of 8 to 10 repetitions at 50% of their perceived maximum load, 3 to 5 repetitions at 75% of their perceived maximum load, and 1 to 3 repetitions at 90% of their perceived maximum load, and after 2 min rest they performed up to 5 maximal attempts. The rest duration between maximal trials was 2 min [28], and the highest valid load lifted was accepted as 1RM. It is important to note that a manual goniometer was used to check joint angles, and that the ICC of the 1RM test was 0.93.

#### 2.4.5. Linear Sprint and CODS Test

Regarding the linear sprint test, athletes must be in the standing position. For this purpose, the toe of the dominant foot will be behind the starting line. The test was started when the athlete voluntarily started the sprint. Single-beam timing gates (Swift Performance Equipment, Lismore, Australia) were used, and the sprint time was assessed to the nearest 0.01 s.

These timing gates were positioned at the start line (0.3 m in front of the subjects), and 20 m from it. Moreover, they were positioned 0.7 m above the floor (i.e., hip level), allowing us to capture the trunk movement only, to avoid a false trigger from one of the limbs.

For the CODS *t*-test, the timing system and procedures were the same as those used in the linear sprint test, except that athletes had to run as quickly as possible while performing several pre-determined CODS [10] and that the testing was performed on an indoor synthetic running surface. Regarding the procedures, before maximal testing, the athletes performed the CODS circuit twice at half speed as a specific warm-up. Thereafter, participants executed three valid maximal attempts, and the rest interval was at least 3 min between each attempt. Note that in the three tests, the best performance was used for the statistical analysis, and the ICC values were 0.94 and 0.88 for the 20 m sprint test and CODS test, respectively. 

#### 2.4.6. Wingate Test

Following previous recommendations [29], anaerobic power was measured with the Wingate anaerobic test, using a computerized Monark Cycle ergometer (Model 894E, Vansbro, Sweden) plus Monark ATS software. So, the seat height was customized for each subject so that the knee angle did not exceed 175 degrees at full extension; additionally, toe clips were used to prevent the feet from slipping off the pedals. Before the test, the athletes pedalled for 5 min at 60–70 revolutions per minute without resistance load. After this warm-up, they had 1 min rest, and the test started with the resistance load adjusted to 0.07 kg per body mass (kg) (mean resistance: 5.0 ± 0.1 kg), in which the athletes should cycle as fast as possible for 30 s. It is an important note that participants were encouraged verbally throughout the test and the peak power (PP) was calculated as the maximum value attained within 5 s during the test.

#### 2.4.7. Cardiorespiratory Endurance

The test was performed on a Motorized Treadmill (Pulsar 3p, H/P Cosmos, Nussdorf-Traunstein, Germany), using a validated protocol [30], in which before the maximal test the athletes performed a specific warm-up involving 3 min walking on the treadmill at 2.75 km/h^−1^ and with a grade of incline of 10%; three minutes later, the speed and slope increased by 1.3 km/h^−1^ and 2%, respectively. This adjustment continued. The test stopped when the athlete could not continue. The athlete’s heart rate (Polar Vantage NV monitor, Polar Electro OY, Kempele, Finland) and the rating of perceived exertion was monitored throughout the test. The output result was cardiorespiratory endurance per minute/second.

### 2.5. Statistical Analyses

Data are presented as group mean ± standard deviation (SDs). After the data normality assumption was verified using the Shapiro–Wilk test, an independent sample *t*-test and two-way mixed analysis of variance (group × time) were performed, with Bonferroni’s *post hoc* test. Partial eta squared (η_p_^2^) was calculated as the effect size of the two-way mixed analysis of variance. When significant differences were noted between groups before the intervention (i.e., DJ height), an analysis of covariance (ANCOVA) was performed, with the pre-test values as the covariate. When the results of both groups were similar, a *t*-test and percentage change were used to compare progress. In addition, Hedge’s *g* effect size was calculated to determine the magnitude of pairwise comparisons for pre- and post-test data, defined as trivial (<0.2), small (≥0.2), moderate (≥0.5), and large (≥0.8) [31]. Statistical calculations were performed with Statistical Package for the Social Sciences (SPSS) version 22.0 and Graph Pad Prism, Model 8.0.1, with a significance level of *p* ≤ 0.05. The sample size was determined by G*Power (G-Power, version 3.1.9.2, University of Dusseldorf, Dusseldorf, Germany) [8]. A statistical power (1-β) of 0.95 was used a priori using the applied comparison analysis (ANOVA for repeated measures, within–between interactions). An effect size of (f = 0.4) and significance level of 5% (*p* < 0.05) were used for all procedures, and the total sample size calculated was *n* = 16, with a 96.49% (actual power) chance of correctly rejecting the null hypothesis of there being no difference in the jump-related biomechanical and physical fitness variable results across time.

## 3. Results

### 3.1. Vertical Jump Height and Related Biomechanical Variables

The jump-related biomechanical variables are indicated in Table 3. There was a significant effect of time on, CMJ RSI (*p* ≤ 0.001, f = 22.41, η_p_^2^ = 0.60), CMJ height (*p* ≤ 0.001, f = 26.16, η_p_^2^ = 0.64), CMJ RFD (*p* = 0.034, f = 5.41, η_p_^2^ = 0.27), and CMJ velocity take-off (*p* ≤ 0.001, f = 19.92, η_p_^2^ = 0.57). There was also a significant group × time interaction for DJ height (*p* = 0.035, f = 5.35, η_p_^2^ = 0.26) and CMJ peak force (*p* = 0.032, f = 5.56, η_p_^2^ = 0.27) in favour of RsG and SsG, respectively. There was no significant effect of time or group × time interaction for DJ RSI, and SJ height.

### 3.2. Physical Fitness

The dependent variables related to physical fitness are indicated in Table 4. There was an effect of time for 20 m sprint (*p* = 0.002, f = 14.45, η_p_^2^ = 0.49), CODS (*p* ≤ 0.001, f = 75.73, η_p_^2^ = 0.84), and PP (*p* ≤ 0.001, f = 78.68, η_p_^2^ = 0.84). A significant group × time interaction was observed for cardiorespiratory endurance (*p* = 0.01, f = 8.03, η_p_^2^ = 0.35) and 1RM leg press (*p* = 0.002, f = 14.07, ηp2 = 0.48), both favouring the SsG.

## 4. Discussion

This study aimed to assess the effects of PJT performed on sand vs. a rigid surface. The major finding was that both surfaces enhanced gains in CMJ time peak force, CMJ RSI, CMJ height, CMJ RFD, CMJ velocity at take-off, 20 m sprint, CODS, and PP in female volleyball players. The CMJ peak force values, as the indicator of muscular strength, at SsG and RsG were revealed, respectively, to be 23.5% and 3.7, which is significant for the SsG. In fact, the CMJ peak force improvement in the SsG was greater compared to the RsG (19.8%). Similar to the CMJ peak force, SsG resulted in greater cardiorespiratory endurance (2.8%) compared with RsG (−0.5%). This outcome demonstrated that SsG increased 1RM (7.8%), whereas RsG revealed a 1.3% improvement; notably, both were significant. The DJ height improved largely after RsG (13.6%) compared with SsG (−3.7%). Overall, the type of surface used during PJT induced specific adaptations in jump-related biomechanical and physical fitness variables among female indoor volleyball players.

Changes in the PJT surface led to adequate overload progress, which serves as a greater stimulus overload than a conventional pattern (more efficacious than manipulating training frequency, intensity, and volume) [8,32]. Therefore, the use of unstable surfaces appears to provide an advantage for athletic performance. In contrast with a previous finding [11] that showed a significant (5.4%) increase in 1RM in a moderate-volume group on a rigid surface compared to a moderate-volume group and high-volume group on a gymnasium surface, our results indicate that *SsG* increased their performance in 1RM (7.8 vs. 1.3%) more than with RsG. Physiologically, the large effect size could be potentially due to recruit the higher motor units of fast-twitch fibres (i.e., IIA and IIX) in the SSC action, with a strong correlation between fast-twitch fibres or the expression percentage of myosin heavy chain II and 1RM having been reported [33,34]. Biomechanically, the qualities of sand absorption allow leg extensor muscles to generate excess prior force in an active state, so the result is an increase in contraction time, which can enable subjects to produce more work on sand vs. a rigid surface, in line with Arazi et al., who reported that the improvement of the 1RM leg press in SsG is greater than RsG in healthy men. Thus, it seems that PJT on sand is likely to cause increases in strength [10]; additionally, functional enhancements in volleyball movements, requiring great strength and fast SSC, were dependent on the training surface [11].

More gains in cardiorespiratory endurance were seen in the SsG than the RsG, compared to the findings of Clemente-Suarez et al. who illustrated that those athletes that have a high aerobic capacity commonly perform higher jumps, which may be related to the coordination of several physical fitness components. This is similar to the results of Hof et al., who measured endurance performance as the time to exhaustion on a ski ergometer at maximum aerobic velocity, after 8 weeks of strength training with an emphasis on neural adaptations in strength and endurance performances in 19 endurance-trained male athletes [35,36]. It has been found that the energetic cost of walking on the sand is more than on rigid surfaces, by engaging more muscle fibres and force to involve the lower extremities (i.e., walking or running) [37]. This might help to explain the greater mechanical work when on sand. The novel approach of this study was using the Bruce test as an explanation of cardiorespiratory endurance. Inasmuch as Bruce test overload is applied and gradually increased (incline and speed) every 3 min, each athlete is able to take part in the test according to their endurance capacity, and it can be interpreted that they have greater cardiorespiratory endurance, according to the formula [30].

The DJ height improved largely for the RsG (13.6%) compared to SsG (−3.7%), whereas for the *SsG*, the improvement (−3.7%) did not reach the required level of significance, which might be related to the increased ground impact force encountered during landing on a rigid surface [1]. Of note, it has been previously found that 7 weeks of PJT on a soft surface (3 cm gym mat) vs. a hard surface (wooden gym floor) induced greater gains (29.7%) in DJ RSI in the hard surface group made up of physically active young men. Given this, a similarly significant increase was observed in DJ20 at either soft surface group (high volume) and hard surface (moderate volume) [11]. Therefore, increasing the PJT volume in the unstable surface than in the rigid surface may help to reach a similar enhancement (due to greater attention being needed to implement PJT over unstable surfaces and the need for more repetitions for adaptation). The greater CMJ height achieved on SsG can be associated with the larger PP needed to augment the transmission power of the muscles on the sand [38]. As the CMJ height can reflect lower-limb muscular strength, a significant positive correlation has been revealed between CMJ height and SJ height [12]. The CMJ velocity take-off on SsG (5.6%) was greater than RsG. A possible interpretation for the effects of PJT on soft surfaces is that it necessitates a potent concentric contraction at push-off, generated by a discharge of elastic energy from the ankle to produce extra energy to overcome the reaction force of the surface [39]. The CMJ take-off velocity induces a sufficient force to jump high, which is explained by the increasing velocity of the employment of motor units in the leg extensor muscles to absorb the eccentric impulse and run-up the concentric impulse [40].

The PP was greater in the SsG compared to RsG (0.3%). This probably occurred because the use of sand gives the opportunity to utilize a greater angular velocity and acceleration at the ankle joint by a larger range of motion; inversely, in the RsG, the interaction of the foot and a firm surface causes ankle stiffness, and so leads to a lower range of motion [23]. It was also noted that improving 1RM could cause a marked improvement of PP [41]. The improvement in PP can be explained by enhancement of CMJ peak force and CMJ velocity as both variables showed significant differences (pre vs. post test) as a correlation probably exists between these variables [42].

In the landing phase of PJT, the mechanical musculoskeletal load was possibly reduced due to the features of the surface; these finding suggest that a double depletion of energy occurs in motor units, and thus muscles are obligated to produce double the force for contraction to overcome the contrary vertical velocity [26]. We assumed that the compliance of the sand is a factor that stimulates the whole body to adjust its state to the onset of vertical take-off. This view indicates that the body attempts to balance the instability of sand with greater hip extension [43]. From the perspective of mechanical variables, the results of vertical ground reaction forces are the main determinants of the movement [44].

In the present study, CMJ RFD levels were distinctly different between groups, increasing performance in the SsG compared to the RsG (27.2 vs. 7.8%). RFD was determined as dynamic aspects of the force and time at maximum voluntary contraction, which in human skeletal muscle is a complex task influenced by numerous methodological factors (i.e., neural, morphological and structural). The influence of neural contractile properties on RFD changes is mainly influenced by the neural drive characteristic in the muscle [26]. Recent evidence suggests the maximal strength and RFD affect CMJ height [41].

Before interpreting the results of this study, it should be taken into consideration that the mechanical work required when on the sand is more than that on hard surfaces [37], supporting the current study’s findings, which illustrated the greater improvement of RFD and CMJ height in SsG, despite the decrease in the efficiency of work carried out by the muscles and tendons through movement on sand in this condition when athlete try to jump as high as they can. This process could strengthen the tendinomuscular system of lower limbs. Therefore, the increases in CMJ height, CMJ peak force, CMJ time peak force, and RFD in the SsG can be explained by changes in the neural drive characteristic, which causes a greater PP by way of faster muscle contractions. However, the PJT regimen improved the athletes’ potential to utilize a significant CMJ peak force level; accordingly, the rationale for the importance of CMJ peak force is known as a key parameter for vertical explosive actions [45]. In accordance with previous findings, it is suggested that the combination of a decreased volume of PJT with a rigid surface may represent an optimal enhancement to implement significant neuromuscular adaptations for sprint and CMJ performance [11].

Both groups increased in terms of CMJ RSI, and the absence of difference in DJ RSI was shown. This could suggest the enhancement of SSC phenomena, which reflects the improvement in CMJ function, since in the vertical jump success is determined by velocity in the take-off phase [42]; under Newton’s formula (F·Δ*t* = m·Δv), each athlete generates a distinct pre-impulse (F·Δ*t*), and the lack of DJ RSI improvement may be explained by the compliance of the sand in which the feet sink and there is an increased contact time, which lead to the exchange of forces generated by the muscles into heat [44,45]. Concerning the DJ RSI, no significant differences were observed in the *RsG* and *SsG*. Although Ramirez-Campillo et al. found a greater DJ RSI in a moderate-volume group on a rigid surface compared to a high-volume group on a soft surface, after 7 weeks of PJT on neuromuscular performance in adolescent men, the moderate-volume group on a rigid surface showed a significant improvement compared to our results; however, the moderate-volume group on a soft surface showed no significant development after the PJT period, which is in line with our study [11]. The 20 m sprint improved in both groups, gains that have been previously reported following 8 weeks of PJT in collegiate rugby players [46]. However, no meaningful enhancement of the 40-yard run time has been demonstrated in amateur football players after PJT on natural and synthetic surfaces [47]. Sprint performance improvements were reported in high-school adolescent men in a high-volume PJT group, as Ramirez-Campillo et al. claimed the manipulation of training volume is more effective than training surface to enhance sprint [11] developments of RFD and has an effect on sprint, which indicates the neuromuscular ability to extend the contraction force through the low level of muscle activation to the end of the performance. This is known as a substantial component of athletes’ strength in most exercises requiring high-speed movement; considering the type of training surface is not efficient to improve 20 m sprint performance [11]. The same is true for SJ; the reversible muscle contractions enable the muscle to produce force, which involves concentric and eccentric phases which play a key role in SSC [40]. It has been shown that the average RFD level contributes 56% of the SPJ performance [12].

The CODS and a quick, short run around the court may be even more important than standing reach height in female players [2]. PJT can stimulate vertically and horizontally oriented force generations, while CODS require a horizontal force, and enhancements in CODS in youth soccer players’ physical fitness have been reported after 7 weeks of PJT [23]. Positive effects of CODS performance with the T-test have also been reported after a PJT intervention on sand compared to the ground in healthy men [10]. It seems that jumping on sand requires a more difficult concentric push-off phase to achieve muscle power, and in line with the present study, further increases in CODS have occurred more in the SsG than RsG after 10 weeks of training in prepubescent girls [48]. Changing the surface may induce larger adaptations after PJT compared with a single training surface [8], and this mechanism can enhance maximal dynamic strength, as the PJT surface, with a moderate volume of training, can present a stimulus to increase explosive performance, requiring fast SSC actions (e.g., CODS), with higher training efficiency [11].

## 5. Conclusions

This study confirmed that 8-week PJT performed on sand and wood training surfaces resulted in some volleyball-related performance changes (biomechanical variables and physical fitness) in female indoor volleyball players.

Therefore, we recommend using sand as a surface, because it appears to induce improvements in CMJ height, CMJ RSI, CMJ peak force, CMJ RFD, CMJ velocity, 20 m sprint, anaerobic power, CODS, cardiorespiratory endurance, and 1RM at equal training loads. Training on a rigid surface, however, improves CMJ height, CMJ RSI, CMJ velocity, 20 m sprint, anaerobic power, CODS, and 1RM.

Given that enhancements are approximately the same for both surfaces, when using different surfaces to supplement conditioning training, it is important to consider the reduction in injury or soreness in athletes. Additionally, the PJT sand surface, as a challenging surface, would acclimatize athletes to a variety (game-like) of reaction ground forces.

## Figures and Tables

**Table 1:** Descriptive data of the SsG and RsG.

Characteristics	SsG (*n* = 8)	RsG (*n* = 9)
Age (y)	23.5 ± 2.8	22.7 ± 2.6
Height (cm)	168.9 ± 4.9	166.5 ± 4.1
Body mass (kg)	59.6 ± 11.3	58.2 ± 7.2
Body mass index (kg/m^2^)	20.7 ± 3.3	21.1 ± 2.6

SsG: sand surface group; RsG: rigid surface group.

**Table 2:** Plyometric jump training program.

Drills	Week							
	1	2	3	4	5	6	7	8
Double-leg hop forward	●	●	●	●	●	●	●	●
Repeated countermovement jump	●	●	●	●	●	●	●	●
Standing single leg hoping	●	●	●	●	●	●	●	●
Double-leg side-to-side jump (30 cm distance)	●	●	●	●	●	●	●	●
Single-leg side-to-side jump (60 cm distance)	●	●	●	●	●	●	●	●
Double-leg hurdle jump forward	●	●	●	●	●	●	●	●
Double-leg depth jump (40 cm high)		●	●	●	●	●	●	●
Tuck jump			●	●	●	●	●	●
Kangaroo jump forward					●	●	●	●
Double-leg box jump (40 cm)							●	●
Ground contacts per session each week	72	84	96	96	108	108	120	120

Each dot demonstrates that relevant drill was performed for 2 sets of 6 repetitions, with an inter-repetition rest of 5–15 s and an inter-set rest of 30–60 s.

**Table 3:** Effects of 8 weeks of plyometric jump training conducted on sand (SsG, *n* = 8) compared to a rigid surface (RsG, *n* = 9) on vertical jump height and related biomechanical variables in female volleyball players.

Variables	Groups	Pre Training	Post Training	Pre-Post (%)	Paired *t*-Test *	Hedge’s *g*
Drop jump height (m)	SsG	0.27 ± 0.05	0.26 ± 0.07	−3.7	0.7	−0.20
	RsG	0.22 ± 0.04	0.25 ± 0.04	13.6	0.01 *	0.70
Drop jump reactive strength index (m/s)	SsG	0.77 ± 0.11	0.74 ± 0.17	−3.9	0.49	−0.20
	RsG	0.61 ± 0.09	0.68 ± 0.19	11.5	0.11	0.40
Spike jump height (cm)	SsG	0.27 ± 0.07	0.26 ± 0.07	−3.7	0.35	−0.10
	RsG	0.25 ± 0.04	0.26 ± 0.04	4.0	0.5	0.20
CMJ reactive strength index (m/s)	SsG	0.42 ± 0.1	0.55 ± 0.08	31.0	0.001 *	1.40
	RsG	0.4 ± 0.11	0.47 ± 0.09	17.5	0.03 *	0.70
CMJ height (cm)	SsG	0.30 ± 0.07	0.35 ± 0.06	16.0	≤0001 *	0.80
	RsG	0.27 ± 0.06	0.30 ± 0.07	11.0	0.04 *	0.46
CMJ peak force (N)	SsG	1179 ± 230	1457 ± 346	23.5	0.002 *	0.90
	RsG	1192 ± 222	1236 ± 261	3.7	0.53	0.20
CMJ rate of force development (N/s)	SsG	1926.98 ± 595.38	2354.72 ± 481.37	22.0	0.04 *	0.90
	RsG	1678.74 ± 326.32	1904.14 ± 443.68	13.0	0.06	0.30
CMJ velocity take off (m/s)	SsG	2.58 ± 0.32	2.95 ± 0.21	12.5	0.002 *	1.30
	RsG	2.52 ± 0.34	2.74 ± 0.27	8.0	0.03 *	0.70

Countermovement jump (CMJ); *: Denote significant pre-post changes at the level of *p* ≤ 0.05.

**Table 4:** Effects of 8 weeks of plyometric jump training conducted on sand (SsG, *n* = 8) compared to a rigid surface (RsG, *n* = 9) on female volleyball players’ physical fitness.

Variables	Groups	Pre Training	Post Training	Pre-Post (%)	Paired *t*-Test *	Hedge’s *g*
20 m sprint (s)	SsG	4.05 ± 0.38	3.96 ± 0.33	−2.2	0.01 *	−0.20
	RsG	4.19 ± 0.27	4.12 ± 0.2	−1.7	0.03 *	−0.30
Wingate peak power (w/kg)	SsG	10.25 ± 1.57	10.48 ± 1.59	2.2	≤0.001 *	0.10
	RsG	10.04 ± 1.48	10.23 ± 1.56	1.9	≤0.001 *	0.10
Cardiorespiratory endurance (min)	SsG	10.26 ± 1.30	10.56 ± 1.38	2.8	0.004 *	0.20
	RsG	9.73 ± 1.22	9.68 ± 1.25	−0.5	0.57	0.04
Change of direction time (s)	SsG	13.33 ± 0.77	12.61 ± 0.83	−5.4	≤0.001 *	−0.80
	RsG	13.61 ± 0.36	13.10 ± 0.38	−3.7	≤0.001 *	−1.30
Leg press one repetition maximum (kg)	SsG	140.55 ± 13.04	151.54 ± 14.08	7.8	≤0.001 *	0.80
	RsG	132.19 ± 12.66	133.97 ± 12.70	1.3	0.31 *	0.10

*: Denote significant pre-post changes at the level of *p* ≤ 0.05.

## Data Availability

Data will be available upon reasonable request to the corresponding author.
